# A Rare Case of Acute Myeloid Leukemia Initially Presenting With Fever of Unknown Origin and Rapidly Progressing Pericardial Effusion

**DOI:** 10.7759/cureus.51505

**Published:** 2024-01-02

**Authors:** Charity Iheagwara, Folasade Ajayi, Onyinye N Otaluka, Carlos Cantu Lopez, Jihad Slim, Hamid S Shaaban, Ala Muhanna, Bereket Tewoldemedhin, Maria Szabela, Jack Boghossian

**Affiliations:** 1 Infectious Diseases, Saint Michael's Medical Center, Newark, USA; 2 Internal Medicine, Saint Michael's Medical Center, Newark, USA; 3 Internal Medicine, University of Nigeria, Enugu, NGA; 4 Hematology and Oncology, Saint Michael's Medical Center, Newark, USA; 5 Internal Medicine, Suburban Community Hospital (Lower Bucks Hospital), Bristol, USA; 6 Infectious Diseases, St. Michael Medical Center, Newark, USA

**Keywords:** fever of unknown origin, post covid pericardial effusion, hemophagocytic lymphohistiocytosis and aml, pericardial effusion and aml, acute myeloid leukemia and fever of undetermined origin, acute myeloid leukemia and pericardial effusion, aml, pericardial effusion, acute myeloid leukemia, fever of undetermined origin

## Abstract

This case report highlights a patient who had persistent fevers for weeks and rapidly progressing pericardial effusion following a positive test for coronavirus disease 2019 (COVID-19) two weeks before presentation to the hospital. The initial thought was that her fever was of infectious etiology, but relevant investigations led to the diagnosis of acute myeloid leukemia (AML). AML, which is characterized by clonal expansion of immature “blast cells” in the peripheral blood and bone marrow resulting in ineffective erythropoiesis and bone marrow failure, is the most prevalent form of leukemia. It is the most aggressive form of leukemia, which has varying prognoses determined by the subtypes. This report explores the association between AML, fever of unknown origin, and pericardial effusion, shedding light on a notable clinical aspect. Fever in AML may be attributed to underlying inflammatory processes, cytokine dysregulation, or bone marrow failure. Recognition of fever as a potential indicator of AML contributes to enhanced clinical vigilance. Pericardial effusions and cardiac tamponade, although rare, can be a presenting feature of AML, and can present side by side with fever of unknown origin as seen in this case report.

## Introduction

Acute myeloid leukemia (AML), which is characterized by clonal expansion of immature “blast cells” in the peripheral blood and bone marrow resulting in ineffective erythropoiesis and bone marrow failure, is the most prevalent form of leukemia, accounting for about 80% of adult leukemias [1]. AML is usually preceded by myelodysplastic syndromes (MDS). MDS manifests as a disorder marked by the excessive proliferation of hematopoietic stem cells, coupled with aberrations in cellular differentiation, leading to peripheral blood cytopenia and the establishment of a preleukemic state [2]. Intriguingly, a notable subset, ranging from 10% to 35%, undergoes progression, evolving into the more aggressive form known as AML through an unclear mechanism [2]. 

In this case report, the diagnosis of AML was complicated by the manifestation of fever of unknown origin (FUO), pericardial effusion, cardiac tamponade, and the fact that the patient tested positive for coronavirus disease 2019 (COVID-19) during her hospital stay. FUO, characterized by persistent fever with an elusive cause, poses challenges in identifying its origin. AML patients may experience febrile episodes categorized as FUO, making it difficult to precisely determine the fever's source. Studies suggest that FUO in the context of AML lacks clearly defined causes, further complicating the diagnostic process [3,4].

Pericardial effusions are not uncommon in patients already diagnosed and being treated for AML (about 21%), but during the initial diagnosis of leukemia, pericardial effusions are rare [5]. This case report highlights a patient who had a persistent fever, a maximum daily temperature that ranged from 100.5℉ to 103.2℉ for over three weeks while in the hospital (FUO), and developed a pericardial effusion and cardiac tamponade prior to being diagnosed with AML. This combination of findings makes this case unique.

## Case presentation

A 45-year-old female with a past medical history significant for type 2 diabetes mellitus and hypertension presented with complaints of retrosternal chest pain and back pain for four days. She acknowledged that her pain started as soreness in the middle of her chest and radiated to her back. Two weeks before her presentation, she tested positive for COVID-19. She experienced mild symptoms, including a fever, but did not seek medical attention.

Physical examination was significant for tachycardia and mild conjunctival pallor. She was awake, alert, and oriented to person, place, and time. Vital signs on arrival at the emergency department were as follows: blood pressure was 122/77 mmHg, pulse rate of 105 beats per minute, respiratory rate of 16 cycles per minute, temperature was 98°F, and oxygen saturation was 97% on room air. Table 1 shows baseline laboratory investigations upon admission.

**Table 1:** Baseline investigations upon admission

Component	Results	Normal value	Units
White Blood Cells	5.0x10^3^	4.4-11x10^3^	u/L
Hemoglobin	7.5	13-17.7	g/dl
Hematocrit	21.7	37.5-51	%
Platelets	45x10^3^	150-450x10^3^	µL
Sodium	135	136-145	mmol/L
Potassium	3.6	3.5-5.3	mmol/L
Chloride	102	98-110	mmol/L
Bicarbonate	23.6	20-31	mmol/L
Blood urea nitrogen	9.0	6-24	mg/dL
Creatinine, serum	0.8	0.6-1.2	mg/dL
Glucose	332	70-140	mg/dL
Aspartate aminotransferase	17	10-36	U/L
Alanine aminotransferase	23	10-49	U/L
Alkaline phosphatase	58	40-116	U/L
High-sensitivity troponin (HS troponin)	113	<34	ng/L
D-dimer	1146	0.0-500.0	ng/ml
Lipase	32	12-53	U/L
Amylase	54	21-101	U/L
International normalized ratio	1.09	0.91-1.1	No unit

Her initial rapid severe acute respiratory syndrome coronavirus 2 (SARS‑CoV‑2) antigen test was negative on admission. An initial respiratory pathogen panel (Table 2) on day five of hospitalization was positive for SARS-CoV-2 and human rhinovirus. A repeat respiratory pathogen panel on day eight of hospitalization was negative for SARS-COV-2.

**Table 2:** Respiratory pathogen panel on day five of hospitalization SARS‑CoV‑2: severe acute respiratory syndrome coronavirus 2; NAA: nucleic acid amplification; PCR: polymerase chain reaction

Component	Results
Adenovirus	Non detected
SARS‑CoV‑2 by NAA	Positive
Human Metapneumovirus	Non detected
Human Rhinovirus/ Enterovirus	Detected
Influenza A PCR	Non detected
Influenza A H1 2009	Non detected
Influenza A H1	Non detected
Influenza A H3 PCR	Non detected
Influenza B	Non detected
Parainfluenza Virus 1	Non detected
Parainfluenza Virus 2	Non detected
Parainfluenza Virus 3	Non detected
Parainfluenza Virus 4	Non detected
Chlamydia pneumoniae	Non detected
Mycoplasma pneumoniae	Non detected
Respiratory syncytial virus A	Non detected
Respiratory syncytial virus B	Non detected
Coronavirus (299E, HKU1, NL63, OC43)	Non detected

**Figure 1:**
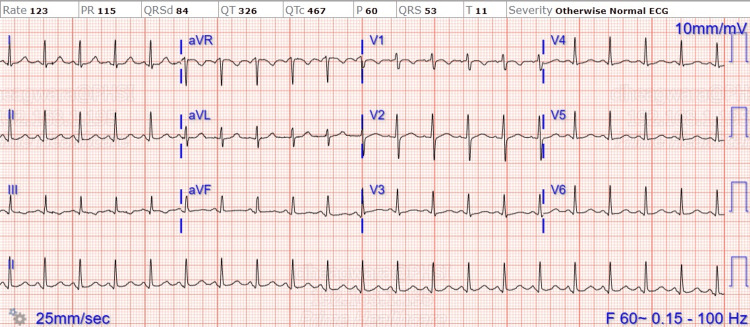
Initial electrocardiogram (EKG) showing abnormal R-wave progression and early transition

Her lactate dehydrogenase was elevated above 750 U/L (normal range: 122-222 U/L), but QuantiFERON Gold (Qiagen, Hilden, Germany), MTB-RIF nucleic acid amplification (NAA) test, and acid-fast bacillus (AFB) smear, and culture of pericardial fluid were negative. Malaria, *Trypanosoma cruzi* antibodies (IgM and IgG), *Mycoplasma*, respiratory syncytial virus (RSV), influenza, adenovirus, herpes simplex virus (HSV) 1 & 2 DNA polymerase chain reaction (PCR) were negative. HIV 1 and 2 antibodies were non-reactive. Antinuclear antibody (ANA), C3, and C4 were within normal limits. Initial and repeat blood cultures remained negative throughout her hospital stay. Pleural fluid fungal culture was negative and pleural fluid cytology was negative for malignant cells. CT pulmonary angiogram ruled out a pulmonary embolism. Her electrocardiogram (EKG) showed abnormal R-wave progression and early transition (Figure 1).

**Figure 2:**
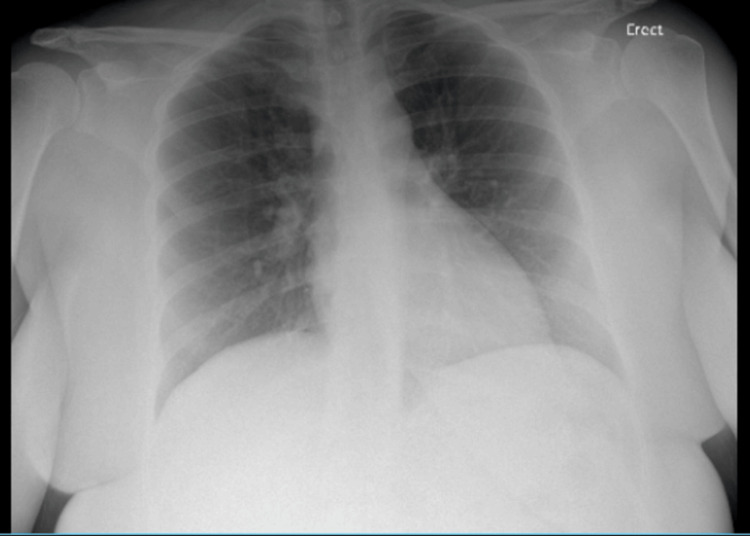
Initial chest X-ray on day one of admission was unremarkable

**Figure 3:**
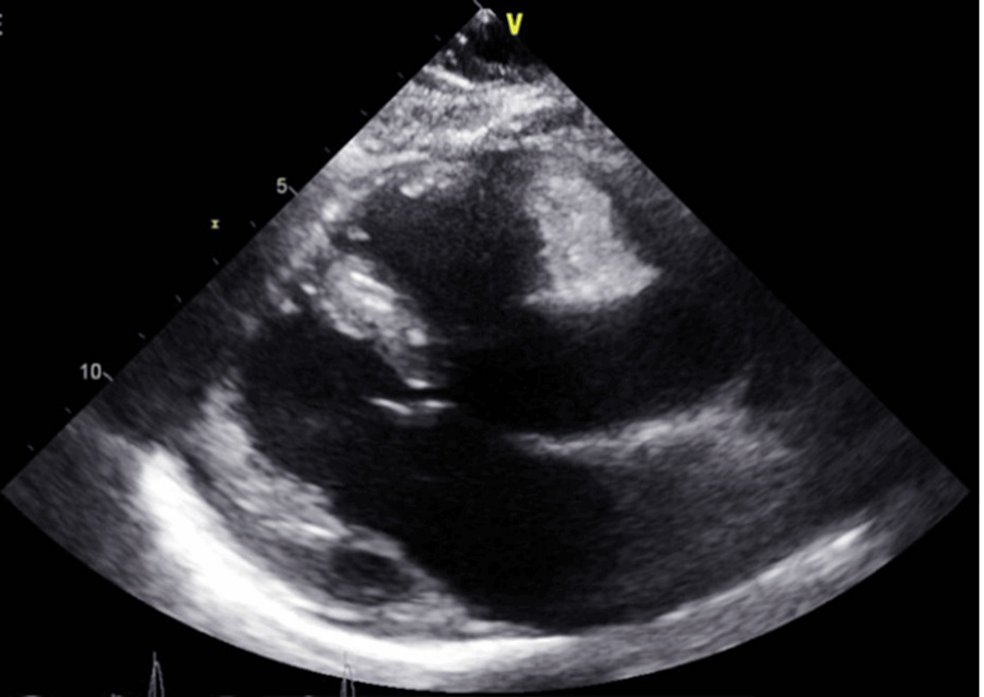
Initial transthoracic echo on admission showing no pericardial effusion

The initial chest X-ray on the day of admission (Figure 2) was unremarkable. An echocardiogram on the day of admission (Figure 3) revealed no pericardial effusion, normal systolic and diastolic function, and left ventricular ejection fraction (LVEF) of 55-60%.

**Figure 4:**
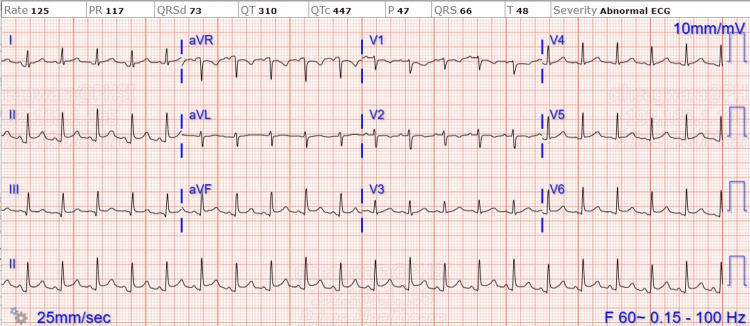
Sinus tachycardia with acute infarct of inferior (left circumflex artery) and lateral leads, with electrical alternans.

A limited echocardiogram was repeated on the second day of admission due to concerns for worsening hypotension and fever and showed moderate-sized pericardial effusion with fibrinous material; no right atrial or ventricular collapse was present. Initial high-sensitivity troponin (HS troponin) was elevated at 113 (normal range: <34 ng/L), and increased to 756 (normal range: <34 ng/L) by day three. On the third day of admission, as the patient's troponin increased, she became hypotensive with a blood pressure of 72/46 mmHg. She was intubated and placed on mechanical ventilation and pressors were initiated. Her EKG showed sinus tachycardia with acute infarct of the inferior (left circumflex artery) and lateral leads, with electrical alternans. (Figure 4).

**Figure 5:**
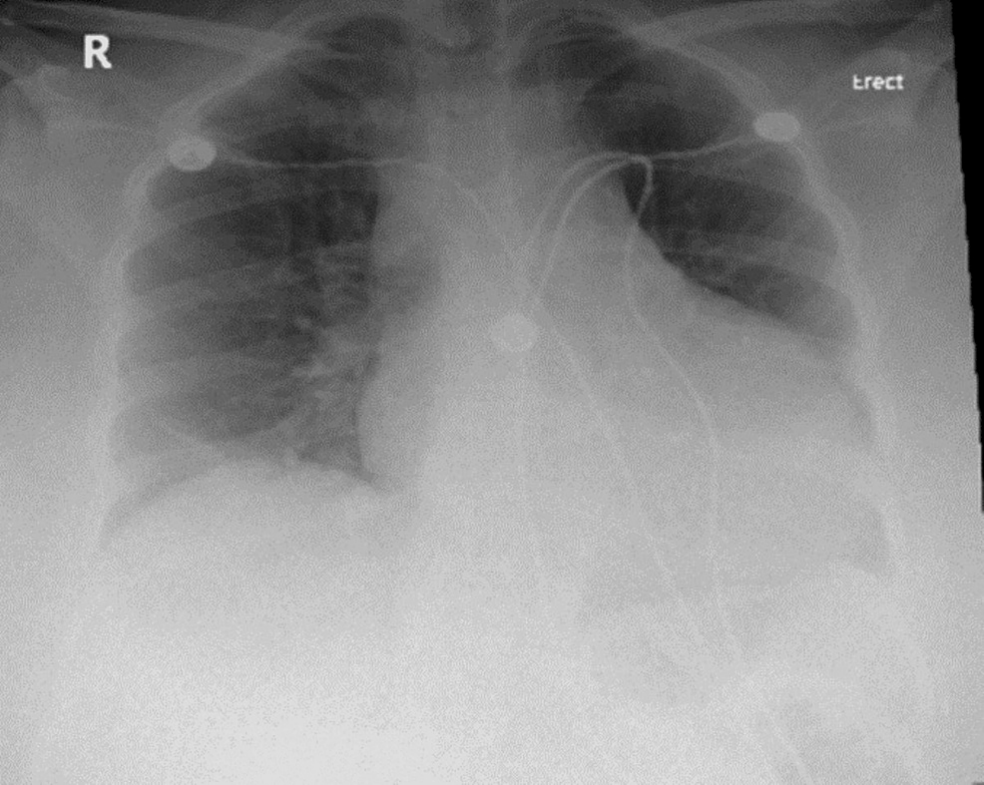
Chest X-ray on day three of admission showing interval cardiomegaly

**Figure 6:**
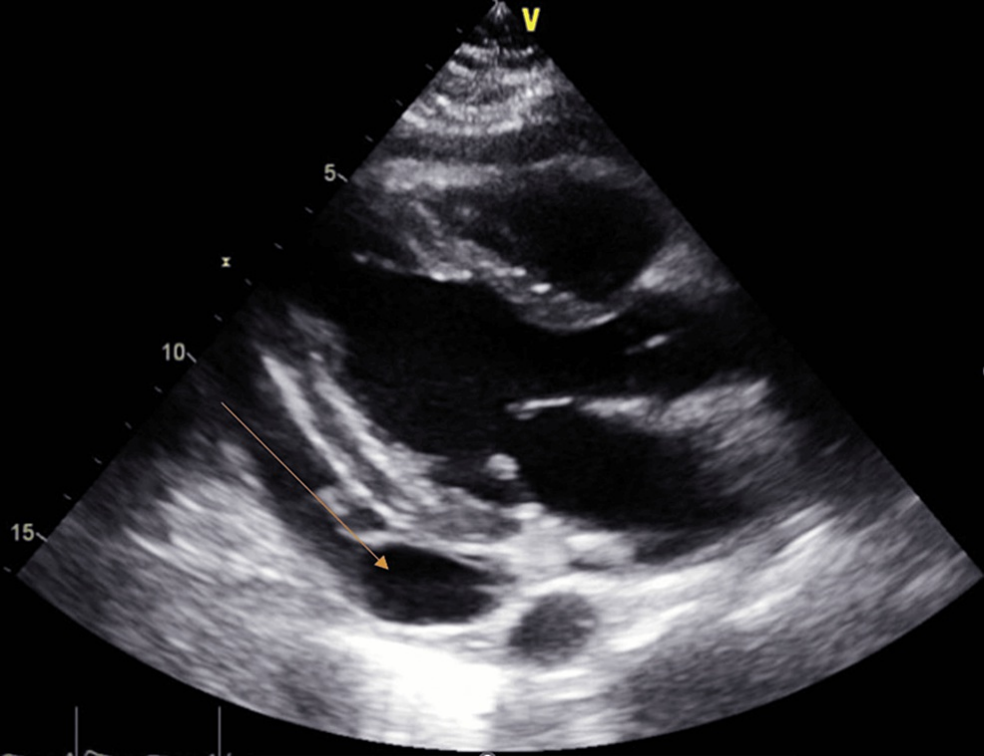
Transthoracic echo on day three showing moderate pleural effusion (orange arrow)

Interval chest X-ray showed cardiomegaly and large bilateral pleural effusions (Figure 5), and chest tubes were placed. Bedside echocardiography was consistent with effusive constrictive pericarditis. A transthoracic echocardiogram on day three showed moderate pleural effusion (Figure 6). The patient developed a rapidly accumulating pericardial effusion from day two of admission and required urgent pericardiocentesis.

The patient underwent pericardiocentesis with drainage of 420 ml of straw-colored pericardial fluid which was sent for appropriate investigations with pericardial fluid culture having no growth. The cytology of pericardial fluid was negative for malignant cells. It showed rare mesothelial cells in a background of inflammatory cells and acellular proteinaceous material. 

She had persistent fever despite coverage with multiple antimicrobial agents for over three weeks. Her maximum daily temperature ranged from 100.5℉ to 103.2℉. Although post-COVID-19 pericarditis could partly explain this clinical presentation, we suspected associated blood dyscrasias in view of significant bicytopenia and persistent fever despite pericardiocentesis and treatment with multiple antibiotics. The patient was unable to mount an appropriate response to the persistent fever given the lack of corresponding leukocytosis as would have been expected during the first few days of admission, the presence of anemia, and worsening thrombocytopenia.

Initial peripheral blood smear showed WBC: occasional atypical lymphocytes and leukocytopenia, RBC: anisocytosis, some microcytic and hypochromic cells, and polychromasia, platelets: rare, enlarged platelets present, thrombocytopenia, and no clumping. Peripheral blood smear on day eleven showed WBC: occasional atypical lymphocytes, a substantial number of immature myeloid cells present and leukocytosis, RBC: anisocytosis, some microcytic and hypochromic cells, polychromasia, platelet: rare, enlarged platelets present, thrombocytopenia, and no clumping, microangiopathic hemolytic anemia was ruled out. She met the laboratory criteria for hemophagocytic lymphohistiocytosis (HLH) with bicytopenia, fever, hypertriglyceridemia, ferritin of 4193 (normal range: 11-307 ng/mL, fibrinogen of 665 (normal range: 200-393 mg/dL, and elevation of soluble CD 25 at 854 (normal range: 79-535 U/mL). High dose steroid was initiated with no clinical response as the fever was persistent. The patient also got a trial of two doses of intravenous immunoglobulins with no clinical response.

**Figure 7:**
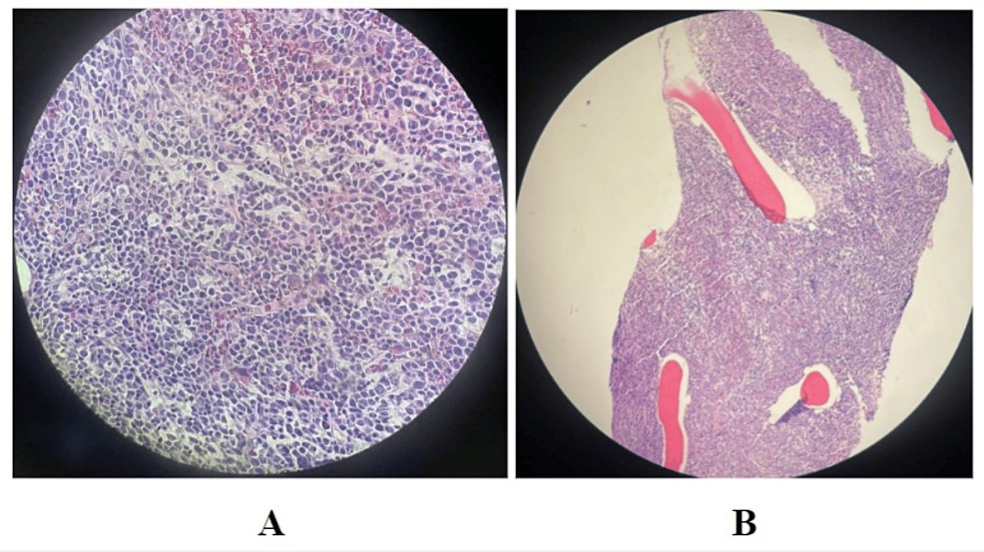
Pathology of bone marrow biopsy showing hypercellular marrow for age (90-100%) with trilineage hematopoiesis.

Peripheral blood flow cytometry showed CD34+ myeloid blasts (5%) and abnormal monocytic cells (monocytes, promonocytes, and monoblasts) (40-60%). Bone marrow biopsy showed hypercellular marrow for age (90-100%) with trilineage hematopoiesis (Figure 7), no granuloma or metastatic carcinoma was seen.

Immunohistochemical stains showed increased CD34 and CD117 positive blasts accounting for 20-30% of bone marrow cellularity. P53 stain was negative. CD138 stains showed increased plasma cells in clusters. AML mutations molecular test and IntelliGeN myeloid test detected NRAS and Tp53 variants. The patient was diagnosed with AML with multilineage dysplasia and monocytic differentiation, pending fluorescence in situ hybridization (FISH). The patient was subsequently transferred to another hospital for further management of her AML, where she was started on idamycin and cytarabine.

## Discussion

AML is a hematologic malignancy associated with various clinical manifestations, including extramedullary involvement. Pericardial effusion, although rare, can be a manifestation of AML and may present with FUO [4]. The association between AML, pericardial effusion, and FUO poses diagnostic challenges and requires a comprehensive medical approach.

AML can infiltrate various tissues, including the pericardium, leading to effusion. Pericardial involvement may result in symptoms such as chest pain, dyspnea, and fever. An FUO can be attributed to the underlying leukemia or associated infections. This can pose a diagnostic challenge as diagnosing pericardial effusion may involve imaging studies like echocardiography, while the workup for FUO includes a thorough examination of potential infectious, neoplastic, and rheumatic causes.

FUO poses a significant challenge in healthcare worldwide, with connections to a wide spectrum of potential diagnoses. The defining criteria include a prolonged axillary temperature of ≥37.8°C observed on multiple occasions, lasting for a minimum of three weeks. When a diagnosis remains elusive after three days of hospital investigation or three outpatient visits, FUO is considered. Infectious, neoplastic, and rheumatic origins are key contributors to this enigmatic condition. Achieving an accurate diagnosis relies on a thorough evaluation of the patient's detailed clinical history and a comprehensive physical examination [6]. This approach then guides the application of specific complementary tests tailored to the unique circumstances of each individual case [6,7]. Our patient had a maximum daily temperature that ranged from 100.5℉ to 103.2℉ for over three weeks while in the hospital.

In an 18-case series, with a sample size of 3164 patients with FUO, 23.2% remained undiagnosed, 11.6% had varying neoplasms, and 37.8% had various infectious diseases that could explain the FUO [8]. Although this patient had a COVID-19 infection two weeks before presentation, it could not explain the persistent fever and sudden deterioration of the patient. In the diagnosis of FUO, clinical judgment is essential as there are various differentials that should be considered which can range from infectious, neoplastic, and rheumatologic etiology. It is important to get a detailed history and conduct a comprehensive physical examination and appropriate laboratory investigations which will include CBC, CMP, CRP, blood cultures, urinalysis and urine cultures, respiratory panel, HIV 1 and 2 serology, ANA, LDH, chest x-ray, CT scan of the chest, abdomen and pelvis, transthoracic echo or transesophageal echo.* Mycobacterium tuberculosis* infections should be ruled out and in patients from endemic regions; malaria and other parasitic infections should be ruled out as well. It is important to have a high index of suspicion and consider getting a bone marrow biopsy to rule out hematologic conditions like MDS and AML.

In the present case, the patient presented with chest and back pain two weeks after she tested positive for COVID-19. She rapidly developed a pericardial effusion and required pericardiocentesis. She had a fever that persisted for weeks despite treatment with various antimicrobial agents and a bone marrow biopsy subsequently revealed a diagnosis of AML. Therefore, it is important to consider obtaining a bone marrow biopsy in cases of FUO [7].

Of note, none of the articles in the literature reviewed noted an initial infection with SARS-CoV-2 prior to diagnosis of AML. Although the pathogenesis of fever in AML remains unclear, most fevers in patients with AML are attributed to infections [8].

In a study involving 747 adults diagnosed with AML, 39.3% (208) presented with FUO despite available wide diagnostic tools for bacterial, fungal, and viral etiology [3,9]. According to the study by Pagano et al., the incidence of FUO among patients with AML is high and remains one of the most challenging issues to be addressed during the evaluation and treatment of immunosuppressed patients [9]. Therefore, it is important to rule out infections that could be responsible. Patients with FUO have a five-year mortality of 3.2% and spontaneous recovery has been reported in 51% of patients [10].

Pericardial effusions rarely present as the initial diagnosis of leukemia although they are thought to be part of the pathogenesis of leukemia due to infectious, hemorrhagic, or leukemic infiltrates, or chemotherapy. It has been associated with some chemotherapy agents used in acute myeloid leukemia, but it is rarely an initial presentation of the disease itself [5]. Pericardial effusion has been reported as a complication or a form of extramedullary metastasis of AML into the heart. Pericardial effusion in AML may arise due to leukemic infiltration into the pericardium, leading to inflammation and fluid accumulation. The involvement of the pericardium can result in symptoms such as chest pain, dyspnea, and a non-specific fever [11].

A study by Sampat et al., which reviewed 1,600 patients already diagnosed with leukemia (acute lymphocytic leukemia (ALL), AML, or MDS) who had an echocardiogram at some point during treatment, showed that pericardial effusion was detected in 21% of 894 patients who had AML [5]. Most of the patients with AML developed pericardial effusion after receiving therapy and not at the initial presentation or prior to initiation of treatment. The difference in survival rates of patients with pericardial effusion and those without did not appear to be significant [5].

Pericardial effusion is more common in patients with underlying malignancy and further management varies from clinical surveillance with echocardiography to pericardial drainage. Pericardial effusions caused by cancer can be managed with the combination of pericardiocentesis or a pericardial window (in cases of recurrent pericardial effusions) in combination with the initiation of antineoplastic agents [5].

In a study involving 93 patients with underlying malignancy, 43 had pericardial effusion of less than 1 cm which did not show progression over time in 96% of them, with similar rates of survival to those who required pericardiocentesis [12]. A total of 29 patients who underwent pericardiocentesis for symptomatic relief showed recurrence of pericardial effusion in 62% of the patients. The study showed that pericardiocentesis provided symptomatic relief in patients with malignancy but did not improve survival and recurrence rates were high.

The bone marrow biopsy results confirming AML with multilineage dysplasia and monocytic differentiation indicated that the patient in the present case also presented with secondary hemophagocytic lymphohistiocytosis (HLH). Secondary HLH is usually associated with malignancies or severe infections, and it is known that patients with AML may be likely to present with HLH due to a susceptibility to severe infections and defective immune response [13]. There remains the question of whether such patients have a genetic defect that predisposes them to the excess immune system activation associated with HLH.

In our patient, the approach was to start induction chemotherapy for AML which led to clinical improvement: resolution of the fevers, improvement in complete blood counts, and no reaccumulating of pericardial effusions requiring drainage.

## Conclusions

This case report underscores the importance of recognizing FUO, pericardial effusion, and cardiac tamponade as relevant clinical features in AML. Clinicians should consider underlying MDS or a possible transformation to AML in the differential diagnosis of unexplained fever, pericardial effusion, and cardiac tamponade. This is crucial for facilitating early intervention and improving patient outcomes. We do recommend a multidisciplinary approach and prompt collaboration with other subspecialties in tackling FUO, with pericardial effusion, and possibly cardiac tamponade to ensure early diagnosis of conditions like AML that need urgent treatment.
